# The effectiveness of spinal cord stimulation combined with physiotherapy in the management of chronic pain in adults: a systematic review

**DOI:** 10.3389/fpain.2025.1620289

**Published:** 2025-07-22

**Authors:** Adilia Maria Soares Porciuncula Barros, Gabrielly Santos Pereira, Josie Resende Torres da Silva, Marcelo Lourenço da Silva, Maria do Desterro da Costa e Silva, Luciano Maia Alves Ferrera

**Affiliations:** ^1^ Alagoas State University of Health Sciences (UNCISAL), Maceió, Brazil; ^2^ Laboratory of Neuroscience, Neuromodulation and Study of Pain (LANNED), Federal University of Alfenas (UNIFAL-MG), Alfenas, Brazil; ^3^ Neuromodulation and Pain Lab (NeuroPain), Egas Moniz Interdisciplinary Research Center (CiiEM), Almada, Portugal

**Keywords:** spinal cord stimulation, chronic pain, neuromodulation, physical therapy modalities, pain management

## Abstract

**Background:**

Chronic pain affects a significant portion of the population, and conventional treatments often prove insufficient. Spinal Cord Stimulation (SCS), a neuromodulation technique, has shown benefits in pain relief, while physiotherapy is widely employed to enhance physical function and quality of life. Although the combination of these approaches may offer synergistic effects, existing evidence is limited and fragmented.

**Objective:**

This systematic review aimed to evaluate the clinical outcomes of Spinal Cord Stimulation (SCS), with or without the association of physiotherapy, in the management of chronic pain in adults. Methodology: The review was conducted following PRISMA guidelines and the PICO strategy. A comprehensive search was performed across databases including Cochrane Library, ScienceDirect, BASE, and VHL (BVS: MEDLINE, IBECS, WPRIM, LILACS, PERIÓDICO CAPES) using MeSH terms and Boolean operators: (“Spinal Cord Stimulation” OR “Neuromodulation”) AND (“Chronic Pain” OR “Pain Management”) AND (“Physical Therapy Modalities” OR “Physiotherapy” OR “Rehabilitation”). Only studies published in English, Spanish, or Portuguese in the past 10 years were included, focusing on chronic pain and reporting outcomes related to pain reduction and functional improvement.

**Results:**

Eight studies comprising 777 patients were included. Spinal cord stimulation alone led to significant pain reductions, with responder rates above 80% and average decreases of 5–6 cm on pain scales. Improvements in quality of life and functional disability were also reported, with reductions of over 30 points in disability indices and up to 40% in opioid use. However, only one study included physiotherapy as a complementary intervention, without isolating its effects. No study directly evaluated the combined efficacy of SCS and physiotherapy, highlighting a gap in the literature.

**Conclusion:**

The findings highlight the proven effectiveness of SCS in chronic pain management but reveal a lack of studies assessing its integration with physiotherapy. Future clinical trials should address this gap to explore potential synergistic effects and optimize interdisciplinary pain treatment strategies.

## 1 Introduction

Chronic pain is a prevalent and debilitating condition that affects a significant portion of the global adult population. Recent estimates suggest that up to 27.5% of individuals worldwide suffer from chronic pain, with prevalence rates varying by region and demographic group (1). This condition exerts a profound impact on patients' quality of life, limiting physical function, affecting mental health, and contributing to significant socioeconomic burdens due to healthcare costs and loss of productivity (2).

**Table 1:** PICO strategy.

P	Adults with chronic pain
I	Spinal Cord Stimulation (SCS) combined with physiotherapy
C	Not applicable
O	Reduction in pain intensity, functional improvement

**Table 2:** Search strategy by database.

Database	Search terms
Cochrane Library	(“Spinal Cord Stimulation” OR “Neuromodulation”) AND (“Chronic Pain” OR “Pain Management”) AND (“Physical Therapy Modalities” OR “Physiotherapy” OR “Rehabilitation”)
BASE	Same as above
Periódicos CAPES	Same as above
BVS – MEDLINE, IBECS, WPRIM, LILACS	Same as above

Conventional management strategies for chronic pain typically include pharmacological interventions—such as non-steroidal anti-inflammatory drugs (NSAIDs), opioids, and antidepressants—as well as non-pharmacological therapies like physiotherapy. However, these approaches often fail to provide sustained relief, particularly in cases involving neuropathic or complex regional pain (3). Moreover, long-term opioid use is associated with the risk of tolerance, dependence, and other adverse effects, prompting the need for safer and more effective alternatives.

Spinal Cord Stimulation (SCS) has emerged as an increasingly utilized modality in the treatment of refractory chronic pain. SCS involves the implantation of electrodes in the epidural space to deliver electrical impulses that modulate pain signal transmission at the spinal cord level (4). This form of neuromodulation has been particularly effective in conditions such as failed back surgery syndrome (FBSS), complex regional pain syndrome (CRPS), and peripheral neuropathies (5).

Technological advancements in SCS, including high-frequency and burst stimulation, as well as closed-loop systems that adapt stimulation in real time, have significantly enhanced clinical outcomes. These innovations provide more consistent pain relief while minimizing side effects like paresthesia, which were common with older stimulation paradigms (6, 7). As a result, SCS is gaining traction as a core component of multimodal pain management strategies.

Parallel to this, physiotherapy remains a cornerstone of chronic pain rehabilitation. It encompasses a variety of techniques—including exercise therapy, manual therapy, patient education, and functional training—designed to reduce pain, restore movement, and improve overall physical performance (8). Physiotherapy emphasizes self-management and long-term function, making it a valuable tool in both early and persistent stages of chronic pain.

The integration of SCS with physiotherapy represents a promising yet underexplored therapeutic approach. The rationale behind this combination lies in the potential for neuromodulation to reduce central sensitization and pain perception, thereby enhancing the patient's ability to engage in rehabilitative exercises and benefit more fully from physiotherapeutic interventions (9, 10). This synergy could lead to better outcomes than either modality alone.

Despite the theoretical and clinical promise of combining SCS and physiotherapy, empirical evidence evaluating their combined effectiveness remains limited. Existing studies often examine each intervention in isolation or include small sample sizes, heterogeneous populations, and variable treatment protocols, making it difficult to draw definitive conclusions.

This review aims to synthesize current evidence regarding pain relief, functional improvement, and quality of life outcomes associated with this integrative approach. The central research question guiding this study is: What is the effectiveness of Spinal Cord Stimulation (SCS) combined with physiotherapy in managing chronic pain in adult patients? The primary objective of this review is to assess the efficacy of this combined intervention in reducing pain and improving functionality. As a secondary objective, the review aims to explore how this combined approach impacts the quality of life in individuals living with chronic pain.

## 2 Methods and materials

This systematic review was conducted to evaluate the effects of combining Spinal Cord Stimulation (SCS) and physiotherapy in the management of chronic pain in adults. In the context of the included studies, physiotherapy was operationalized as non-invasive physical rehabilitation strategies, encompassing exercise-based interventions, motor training, and functional reconditioning programs aimed at improving mobility, reducing disability, and enhancing quality of life. The review followed the PRISMA (Preferred Reporting Items for Systematic Reviews and Meta-Analyses) guidelines to ensure transparency and reproducibility throughout the process. Additionally, the PICO strategy was used to structure the research question and guide the study selection (Table 1): P (Population): Adults with chronic pain; I (Intervention): Spinal Cord Stimulation (SCS) combined with physiotherapy; C (Comparison): Not applicable, as the review focused on describing the effects of a combined intervention without direct comparison to another approach;O (Outcome): Reduction in pain intensity and improvement in physical functionality. Accordingly, the guiding research question was: “What is the effectiveness of Spinal Cord Stimulation (SCS) combined with physiotherapy in managing chronic pain in adult patients?”

A comprehensive literature search was conducted across multiple academic databases (Table 2), including Cochrane Library, ScienceDirect, Bielefeld Academic Search Engine (BASE), and CAPES Journal Portal (Periódicos CAPES). Through the Virtual Health Library (BVS), the following databases were also accessed: MEDLINE, IBECS, WPRIM, and LILACS. The search was based on MeSH (Medical Subject Headings) terms: “Spinal Cord Stimulation”, “Chronic Pain”, “Neuromodulation”, “Physical Therapy Modalities”, and “Pain Management”. Boolean operators AND and OR were used to combine terms as follows:

Search strategy was: (“Spinal Cord Stimulation” OR “Neuromodulation”) AND (“Chronic Pain” OR “Pain Management”) AND (“Physical Therapy Modalities” OR “Physiotherapy” OR “Rehabilitation”).

Inclusion criteria consisted of studies published in the last ten years, written in English, Spanish, or Portuguese, involving adult patients (≥18 years old) diagnosed with chronic pain, investigating the combined use of SCS and physiotherapy (in any of its forms), and reporting outcomes related to pain reduction and physical function improvement.

Exclusion criteria included studies involving children or adolescents, those that did not address the primary objective, review articles, editorials, studies without quantitative data, those focused exclusively on acute pain or immediate postoperative recovery, and duplicate studies (identified using the Rayyan tool).

The article selection process involved two phases: first, screening of titles and abstracts to identify relevant studies; second, full-text analysis of the selected articles according to the eligibility criteria. Extracted data were organized into tables emphasizing chronic pain management and functional outcomes. The methodological quality of the included studies was assessed using the Cochrane Collaboration's risk of bias tool.

The primary objective of this review was to assess the effectiveness of Spinal Cord Stimulation combined with physiotherapy in managing chronic pain and improving physical functionality. The secondary objective was to explore how this combined intervention influences the quality of life in individuals living with chronic pain.

## 3 Results

**Figure 1:**
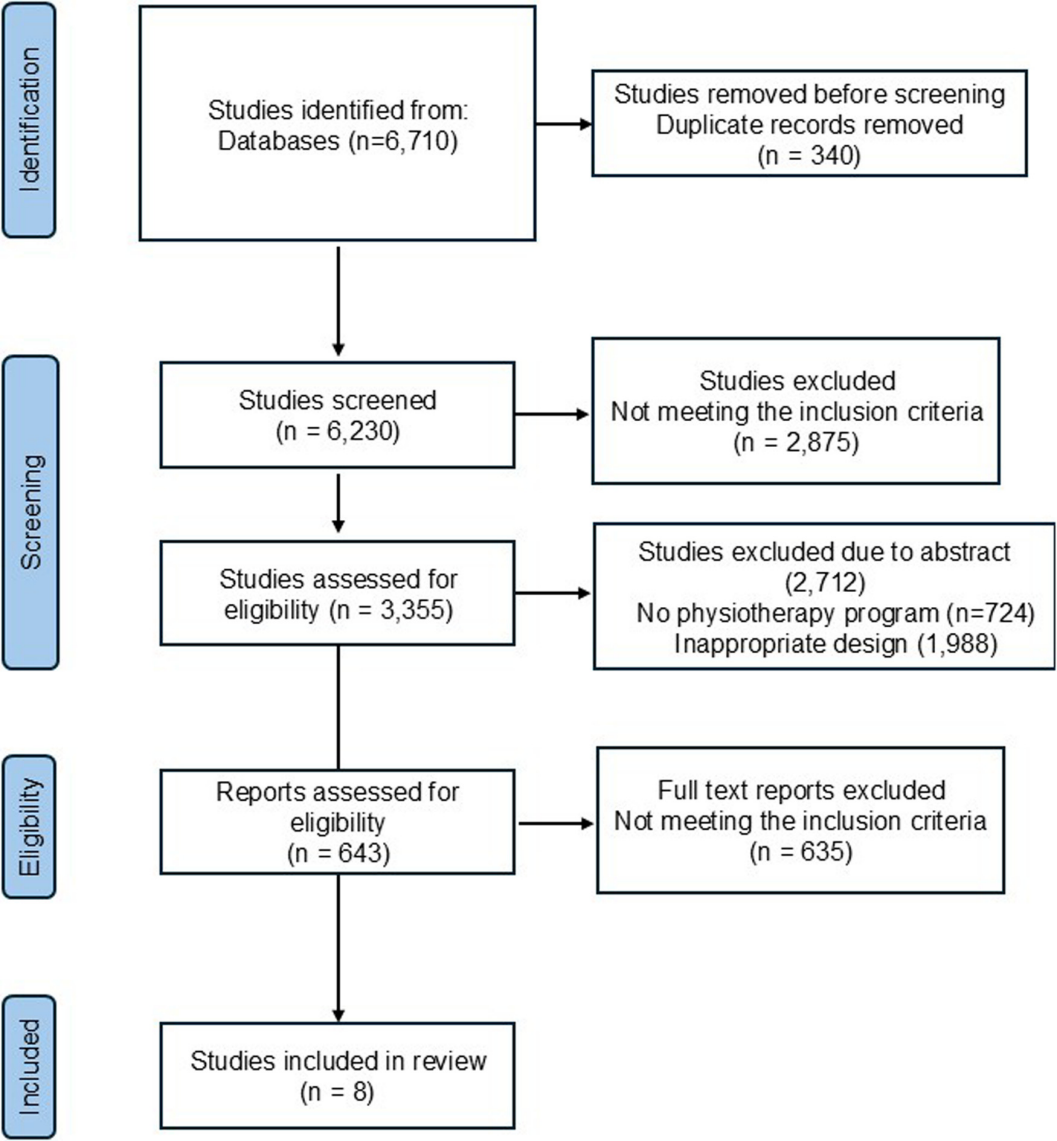
PRISMA flow diagram.

The PRISMA flow diagram illustrates the article selection process (Figure 1). Initially, 6,710 records were identified from various databases, including the Cochrane Library, ScienceDirect, BASE, Capes Journals, BVS, MEDLINE, IBEC, WPRIM, and LILACS. After removing 340 duplicate records, 6,370 articles were screened. Of these, 3,568 were excluded for not meeting the inclusion criteria—2,110 due to being outside the five-year time frame and 1,458 for not aligning with the thematic scope of the study. The remaining 2,802 records were screened by title and abstract, resulting in the exclusion of 2,682 records. A total of 123 full-text publications were assessed for eligibility, of which 114 were excluded for not meeting the inclusion criteria. Ultimately, 8 studies were included in the systematic review.

The analysis of the selected studies presented in Table 3 reveals a consistent trend in favor of the use of spinal cord stimulation (SCS), especially when combined with physical therapy, for the management of chronic pain.

**Table 3:** Results of article search and selection.

Author	Sample	Title	Type of Study	Methodology	Outcomes	Results
AHMADI, et al. (11)	8 patients (surgery-naïve; ineligible for lumbar surgery)	*High-Frequency Spinal Cord Stimulation in Surgery-Na€ıve Patients—A Prospective Single-Center Study*	Prospective cohort study	Patients with chronic low back and/or leg pain underwent high-frequency SCS trial (10-kHz) for at least one week. Permanent implantation followed for responders. Programming was adjusted if pain persisted. Follow-up mean: 306 days (∼10 months).	Daily pain intensity (numeric rating scale – NRS) for back and leg pain; adverse events	NRS for back pain: reduced from 8.9 ± 0.23 to ∼4.8 → *Δ* = −4.1 NRS for leg pain: reduced from 8.1 ± 0.6 to ∼1.9 → *Δ* = −6.2 No formal statistical analysis or CIs reported due to small sample 2 patients had IPG site irritation; both revised surgically; no infections
AL-KAISY, et al. (12)	21 enrolled; 20 implanted; 17 completed 36-month follow-up	*Long-Term Improvements in Chronic Axial Low Back Pain Patients Without Previous Spinal Surgery: A Cohort Analysis of 10-kHz High-Frequency Spinal Cord Stimulation over 36 Months*	Prospective, open-label clinical study	Patients with chronic axial low back pain and no prior spine surgery underwent trial stimulation with 10-kHz HF-SCS. Those with ≥50% pain relief received permanent implants. Outcomes were tracked over 36 months.	VAS for back pain, Oswestry Disability Index (ODI), opioid use	From baseline to 36 months: VAS decreased from 79 ± 12 to 10 ± 12 mm → *Δ* = −69 mm ODI improved from 53 ± 13 to 19.8 ± 13 → *Δ* = −33.2 points Opioid use: 18 patients at baseline vs. 2 at 36 months No statistical tests or CIs reported, but magnitude of change is clinically significant
GULISANO, et al. (14)	16 patients with chronic pancreatitis and refractory visceral pain	*A sham-controlled, randomized trial of spinal cord stimulation for the treatment of pain in chronic pancreatitis*	Randomized, double-blind, sham-controlled, crossover trial	Participants received either high-frequency (1,000 Hz) paraesthesia-free SCS or sham stimulation for two 10-day periods, separated by a 3-day washout. Pain was assessed using daily numeric rating scales (NRS). Secondary outcomes included quality of life and sensory testing. After the blinded phase, patients entered an open-label 12-month extension.	Primary: Average daily pain intensity (NRS) Secondary: Maximal daily pain score, quality-of-life questionnaires, quantitative sensory testing	No significant difference between SCS and sham in NRS pain scores: Mean difference = −0.1; 95% CI [−1.4 to 1.1]; *P* = 0.81 Open-label phase showed significant long-term improvement in NRS: From 5.2 ± 1.7 at baseline to 2.9 ± 1.9 at 6 months (*P* = 0.001) No significant differences in secondary outcomes or QST in blinded phase
KALLEWAARD, et al. (15)	112 participants randomized (DTM SCS: 55; CMM: 57)	*European randomized controlled trial evaluating differential target multiplexed spinal cord stimulation and conventional medical management in subjects with persistent back pain ineligible for spine surgery: 24-month results*	Prospective, multicenter, open-label randomized controlled trial (with optional crossover)	Patients with chronic low back pain (CLBP) and no prior spine surgery (PSPS-T1) were randomized 1:1 to receive Differential Target Multiplexed SCS (DTM SCS) or conventional medical management (CMM). Primary endpoint was the proportion of responders (≥50% CLBP reduction) at 6 months. Secondary measures included leg pain, functional disability, quality of life (QoL), opioid intake, and a Composite Responder Index (CRI). Patients were followed for 24 months.	Primary: ≥ 50% CLBP reduction (NRS) Secondary: CRI (pain, ODI, EQ-5D), opioid consumption, PGIC, satisfaction, leg pain scores	At 6 months, CLBP responder rate was 80.4% (DTM SCS) vs. 11.1% (CMM). Risk difference = 69.3%; 95% CI [53.9–84.8]; *P* < 0.001 CRI ≥80% in DTM SCS group at 6, 12, and 24 months Mean pain relief: 5.8 ± 2.2 cm at 6 months; 6.1 ± 2.4 cm at 24 months Opioid reduction significantly greater in DTM SCS group (*P* = 0.008) PGIC “very much improved/much improved”: 89% in DTM SCS vs. 15.8% in CMM AE incidence was low and comparable to prior SCS studies
KAPURAL, et al. (6)	198 randomized; 171 implanted (88 HF10, 83 traditional SCS)	*Novel 10-kHz High-frequency Therapy (HF10 Therapy) Is Superior to Traditional Low-frequency Spinal Cord Stimulation for the Treatment of Chronic Back and Leg Pain* *The SENZA-RCT Randomized Controlled Trial*	Randomized controlled trial (parallel-arm, noninferiority)	Multicenter RCT comparing 10-kHz high-frequency SCS (HF10) vs. traditional low-frequency SCS in patients with chronic back and leg pain. All patients underwent trial stimulation; responders (≥50% pain reduction) received permanent implants. Follow-up extended to 12 months.	Primary: ≥ 50% back pain reduction (responders). Secondary: Responder rate for leg pain, maintenance of effects, and paresthesia occurrence.	At 3 months: Back pain responders: HF10 = 84.5% vs. Traditional = 43.8%. RR = 1.9; 95% CI [1.4–2.5]; *P* < 0.001. Leg pain responders: HF10 = 83.1% vs. Traditional = 55.5%. RR = 1.5; 95% CI [1.2–1.9]; *P* < 0.001 HF10 superiority persisted through 12 months; no paresthesias in HF10 group.
ELKHOLY, et al. (13)	74 patients with Failed Back Surgery Syndrome (FBSS) who received spinal cord stimulation (SCS)	*Effect of spinal cord stimulation on quality of life and opioid consumption in patients with failed back surgery syndrome*	Prospective observational cohort study	Patients with FBSS treated with SCS at a single center in Germany between 2010 and 2021 were prospectively assessed. Quality of life (QoL) and opioid consumption were evaluated at baseline and during follow-up at 6, 12, 24, and 36 months. Instruments included the EQ-5D and EQ-VAS, as well as analysis of prescribed opioid dosages converted into morphine equivalents (MEQ/day).	Primary: EQ-5D utility score and EQ-VAS. Secondary: Daily opioid consumption (mg MEQ), pain scores, and patient satisfaction	EQ-5D utility score: increased from 0.31 ± 0.31 at baseline to 0.55 ± 0.28 at 6 months (*Δ* = 0.24; *P* < 0.001) and maintained up to 36 months EQ-VAS: improved from 37.1 ± 18.1 to 56.4 ± 20.4 at 6 months (*Δ* = 19.3; *P* < 0.001) Opioid use: decreased from 82.6 ± 65.6 mg MEQ/day at baseline to 49.3 ± 47.2 mg at 6 months (*P* < 0.001) Sustained opioid reduction and QoL improvements observed at 12, 24, and 36 months No severe adverse events related to SCS reported
RIGOARD, et al. (16)	218 patients with Failed Back Surgery Syndrome (FBSS) and predominant back pain (SCS group: *n* = 110; OMM group: *n* = 108)	*Multicolumn spinal cord stimulation for predominant back pain in failed back surgery syndrome patients: a multicenter randomized controlled trial*	Multicenter, randomized, open-label, parallel-group controlled trial	FBSS patients with predominant low back pain were randomized 1:1 to receive multicolumn spinal cord stimulation (SCS) plus optimal medical management (OMM) or OMM alone, across 28 international centers. Patients in the SCS arm underwent a trial phase, followed by permanent implantation upon successful response. Primary outcome was assessed at 6 months, with additional follow-ups at 1, 3, 6, and 12 months. Patients were allowed to cross over after 6 months.	Primary: Proportion of patients with ≥50% reduction in low back pain (NRS) at 6 months Secondary: Pain intensity (NRS), functional disability (ODI), health-related quality of life (EQ-5D), opioid use, adverse events	Primary outcome (6-month responders): SCS group = 13.6% (15/110) vs. OMM = 4.6% (5/108). Risk difference = 9.0%; 95% CI [0.6–17.5]; *P* = 0.036. Secondary outcomes: Significant improvement in pain (back and leg), ODI, and EQ-5D in the SCS group. Adverse events in SCS group: 17.6% (18/102), with 11.8% requiring reintervention
WILL et al. (17)	50 participants (Closed-loop ECAP-controlled SCS: *n* = 26; Open-loop SCS: *n* = 24)	*Improvements in Therapy Experience With Evoked Compound Action Potential Controlled, Closed-Loop Spinal Cord Stimulation—Primary Outcome of the ECHO-MAC Randomized Clinical Trial*	Multicenter, randomized, double-blind, parallel-group controlled trial	Participants with chronic trunk and/or limb pain were randomized 1:1 to receive closed-loop spinal cord stimulation (CL-SCS)—which modulates stimulation based on real-time ECAPs—or open-loop stimulation (OL-SCS). The primary outcome was the improvement in therapy experience [assessed by the Pain Experience Questionnaire–Patient Reported (PEQ-PR)] at 3 months. Secondary outcomes included pain intensity (NRS), global impression of change (PGIC), quality of life (EQ-5D), and sleep disturbance.	Primary: Change in PEQ-PR Total Score (therapy experience) Secondary: NRS pain, PGIC, EQ-5D, sleep, emotional well-being, and adverse events	PEQ-PR Total Score improvement at 3 months: CL-SCS: + 34.1 vs. OL-SCS: + 18.1. Mean difference = + 16.0; 95% CI [3.3–28.7]; *P* = 0.014 Higher proportion reporting “very much improved/much improved” in CL-SCS group (80.8% vs. 41.7%; *P* = 0.007). Sleep and emotional well-being also significantly improved in CL-SCS No severe device-related adverse events reported.

Summary of included studies evaluating the effects of spinal cord stimulation (SCS) for chronic pain management across different patient populations. The table details the study authors, sample characteristics, titles, type of study, methodological approaches, outcome measures assessed, and main results—including effect sizes, confidence intervals (CIs), and statistical significance where reported.

AE, adverse event; CIs, confidence intervals; CMM, conventional medical management; CLBP, chronic low back pain; CL-SCS, closed-loop spinal cord stimulation; DTM SCS, differential target multiplexed spinal cord stimulation; ECAP, evoked compound action potential; EQ-5D, euroQol-5 Dimensions (measure of health-related quality of life); EQ-VAS, euroQol visual analogue scale; FBSS, failed back surgery syndrome; HF10, 10-kHz high-frequency spinal cord stimulation; IPG, implantable pulse generator; LBP, low back pain; MEQ, morphine equivalent dose; NRS, numeric rating scale; ODI, oswestry disability index; OL-SCS, open-loop spinal cord stimulation; OMM, optimal medical management; PEQ-PR, pain experience questionnaire – patient reported; PGIC, patient global impression of change; PSPS-T1, persistent spinal pain syndrome type 1 (patients without prior spine surgery); QoL, quality of life; RCT, randomized controlled trial; SCS, spinal cord stimulation; VAS, visual analogue scale.

### 3.1 Risk of bias assessment

**Figure 2:**
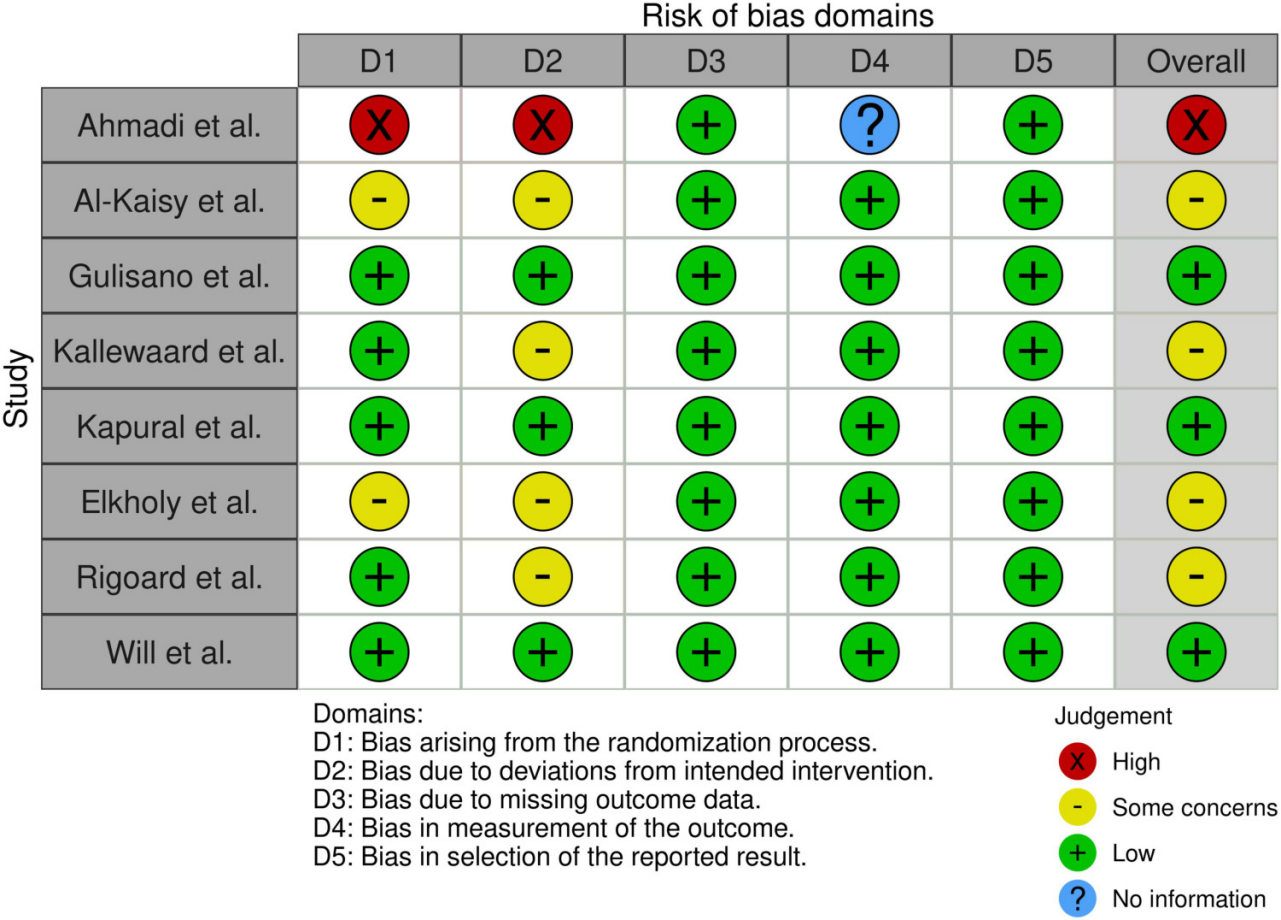
Risk of bias assessment.

The risk of bias across the eight selected studies was evaluated using a domain-based approach adapted from the Cochrane Risk of Bias tool, and the results are summarized in Figure 2. The domains assessed included selection bias, performance bias, detection bias, attrition bias, and reporting bias.

Overall, the majority of studies presented low risk of detection and reporting bias, indicating that outcome measures were clearly defined and reported with transparency. However, moderate to high risk of bias was observed in the domains of selection and performance, particularly in non-randomized studies such as Ahmadi et al. (11), Al-Kaisy et al. (12) and Elkholy et al. (13), where the absence of random allocation and blinding of participants and personnel may have introduced systematic error.

Attrition bias was generally low across studies, except for Ahmadi et al. (11), which showed unclear risk due to limited information regarding follow-up completeness. The only sham-controlled, double-blind trial (14) demonstrated low risk in all domains except performance bias, owing to the challenge of maintaining participant blinding beyond the short-term intervention phase.

Randomized controlled trials such as those by Kallewaard et al. (15), Kapural et al. (6), Rigoard et al. (16), and Will et al. (17) displayed low to moderate risk across all domains, enhancing the reliability of their findings. Nevertheless, despite methodological robustness in some cases, variability in study designs, small sample sizes, and absence of pre-registered protocols in certain studies highlight limitations in internal validity and comparability. These findings reinforce the need for larger, methodologically rigorous RCTs with standardized protocols and longer follow-up to strengthen the evidence base on spinal cord stimulation for chronic pain.

## 4 Discussion

From an epidemiological perspective, chronic pain is more prevalent in women and middle-aged adults, often exacerbated by social and cultural factors such as limited access to effective treatments in certain populations. Strategies for managing this condition include multidisciplinary approaches that combine medical treatment, psychological support, and lifestyle interventions, such as physical activity and sleep improvement practices (18, 19).

The analysis of the seven selected articles reveals consistent and promising evidence supporting the effectiveness of spinal cord stimulation (SCS) in the management of chronic pain. Gulisano et al. (14) and Kallewaard et al. (15) demonstrated in randomized controlled trials that SCS significantly reduces pain and enhances quality of life in patients with chronic pancreatitis and persistent back pain, respectively, with sustained benefits observed over 24 months in the latter. Elkholy et al. (13) further corroborated these findings, showing improvements in both quality of life and decreased opioid consumption in patients with failed back surgery syndrome. Will et al. (17) reinforced the therapeutic advantages of closed-loop SCS, highlighting enhanced patient experiences and greater functional improvement. Collectively, these studies underscore the growing body of literature favoring SCS as an effective intervention for various forms of chronic pain, while also emphasizing the need for further long-term and comparative research to optimize clinical outcomes.

In addition to these studies, the high-frequency 10-kHz modality of SCS has emerged as a particularly promising approach. Kapural et al. (6), in a landmark randomized controlled trial (SENZA-RCT), demonstrated that HF10 therapy was significantly superior to conventional low-frequency SCS for the treatment of chronic back and leg pain, with greater responder rates and sustained efficacy over 12 months, without inducing paresthesias. Al-Kaisy et al. (12) extended this evidence by showing long-term efficacy of 10-kHz SCS in a surgery-naïve population with axial low back pain: patients experienced marked and sustained reductions in pain and disability, as well as decreased opioid use, over a 36-month follow-up. Ahmadi et al. (11) further supported these outcomes in a cohort of inoperable patients, reinforcing the role of high-frequency SCS in cases where traditional surgical options are not viable. Together, these findings expand the applicability of SCS beyond post-surgical pain and highlight its potential for broader chronic pain populations.

Chronic pain involves a multifactorial process encompassing changes in both the peripheral and central nervous systems, particularly peripheral and central sensitization phenomena. Peripheral sensitization occurs due to the repeated activation of nociceptors, typically linked to inflammatory conditions or injuries. Continuous exposure to inflammatory mediators—such as prostaglandins, bradykinins, and cytokines—lowers the activation threshold of sensory nerves, making them more likely to transmit pain signals even in response to minimal stimuli. This results in hyperalgesia, where mild stimuli are perceived as painful (20, 21).

Central sensitization, on the other hand, occurs at the level of the central nervous system, mainly in the dorsal horn of the spinal cord and brain structures like the thalamus and somatosensory cortex. Prolonged exposure to pain signals activates NMDA (N-methyl-D-aspartate) receptors, increasing neuronal excitability and decreasing the effectiveness of descending inhibitory systems. This state perpetuates pain even after the initial stimulus has resolved. Glial cell activation—particularly astrocytes and microglia—plays a key role by releasing pro-inflammatory substances that amplify pain signaling (9, 22).

In addition, the concept of “nociplastic pain” has emerged as a third category of pain, present in conditions such as fibromyalgia and irritable bowel syndrome. In this case, there is no apparent tissue damage or injury to the somatosensory system, but a dysregulation in pain processing, characterized by altered descending control and increased central sensitivity (23).

Chronic pain is also associated with psychological and emotional changes, such as anxiety and depression, which often exacerbate pain perception. The interaction between the nervous, endocrine, and immune systems contributes to a continuous cycle of pain and stress. These biopsychosocial changes make chronic pain management complex, requiring multidisciplinary interventions that include pharmacotherapy (such as antidepressants and anticonvulsants), physiotherapy, and cognitive behavioral therapy. Innovative approaches like neuromodulation and mindfulness have shown efficacy in modulating central pain processing (24, 25).

Beyond physical recovery benefits, spinal cord stimulation (SCS) has also shown a positive impact on reducing the use of analgesic medications, especially opioids. Long-term use of these drugs is often associated with serious side effects, including dependence, making the combination of SCS and physiotherapy even more appealing as it offers a non-pharmacological alternative for pain management (14). The pain relief provided by SCS may enable patients to engage in rehabilitation activities that would be difficult or impossible while experiencing severe pain, such as strengthening and stretching exercises. This not only improves mobility but may also positively affect the patient's mental health, promoting a sense of well-being and control over their condition.

Although the initial results of this therapeutic combination are promising, successful implementation of an SCS and physiotherapy protocol requires a personalized approach and careful monitoring. Patients with complex chronic pain, such as those with failed back surgery syndrome, may have specific needs that demand adjustments in therapy intensity or physiotherapeutic approach. Physiotherapy must be tailored to the patient's pain level and stage of recovery, as performing intense exercises without adequate pain modulation may be counterproductive (26). Therefore, collaboration among physiotherapists, pain specialists, and neurosurgeons is essential to ensure that the treatment plan is optimized for each patient, considering their individual needs.

Moreover, it is important to recognize that SCS, while effective in many cases, may not be suitable for all chronic pain patients. Those with specific contraindications, such as blood clotting disorders or chronic infections, may not be candidates for SCS implantation. In such cases, physiotherapy can serve as an alternative or complementary approach for pain reduction without surgical intervention (27). Thus, personalized treatment becomes even more important, and physiotherapy remains a fundamental, non-invasive therapy with few associated risks and the potential to provide long-term benefits in pain and function management.

Given this context, the combination of SCS and physiotherapy presents a promising strategy for chronic pain management, with the ability to provide immediate symptomatic relief through electrical stimulation while promoting functional recovery and long-term quality of life improvements (28). Although early results are encouraging, further long-term studies are necessary to confirm the sustainability of benefits from this combined approach. Personalized treatment with a rigorous follow-up protocol is crucial to maximize benefits and ensure patient safety while minimizing the risks associated with implementing these therapies.

Spinal cord stimulation (SCS) is a minimally invasive technique used to treat chronic pain, especially in patients with neuropathic pain. The procedure involves inserting electrodes near the spinal cord to deliver electrical impulses that modulate nerve activity and block the perception of pain. This method is particularly recommended for patients who do not respond to conventional or surgical treatments (29). The process is usually divided into several stages, beginning with careful patient selection, during which factors such as pain duration, medical history, and previous treatment failures are assessed.

Following this, a preliminary SCS trial is conducted, involving the temporary insertion of a percutaneous stimulation system. This trial aims to assess the patient's response to treatment, adjusting the intensity and frequency of electrical impulses as needed. If the trial is successful, the procedure proceeds with the permanent implantation of the device, which consists of an electrical pulse generator placed under the patient's skin—typically in the lumbar or abdominal region—and connected to the electrodes positioned near the spinal cord (30).

After implantation, the device is programmed to deliver electrical impulses, with adjustments to intensity, frequency, and pulse width to optimize treatment. Continuous monitoring is essential to adjust the device parameters, ensure treatment efficacy, and maintain therapeutic effects. SCS has proven to be an effective solution for patients with chronic pain, especially neuropathic pain, and is supported by numerous scientific studies (31).

SCS offers a treatment alternative when conventional approaches fail, providing significant pain relief in many patients. Thus, SCS is established as an effective alternative in chronic pain management, offering a personalized approach aimed at optimizing pain relief and improving patient quality of life (32).

The association between spinal cord stimulation (SCS) and physiotherapy is essential for the effective treatment of chronic pain patients, especially those who benefit from SCS-mediated pain modulation. Combining these two therapeutic approaches can significantly enhance clinical outcomes, as physiotherapy aims to restore physical function and mobility, while SCS helps control pain (33). However, there are significant challenges to the effective integration of physiotherapy and SCS, ranging from technical limitations to issues related to pain treatment itself.

Spinal cord stimulation, by delivering electrical impulses that modulate pain, can reduce chronic pain perception, enabling patients to participate more actively in physical rehabilitation programs. Physiotherapy, in turn, aims to improve muscle strength, flexibility, coordination, and endurance, as well as joint functionality and postural control. The combination of SCS and physiotherapy can result in faster and longer-lasting functional recovery since reduced pain allows patients to engage more fully in prescribed physical activities and exercises (34).

SCS has shown a positive impact on pain reduction in patients with chronic low back pain, facilitating the implementation of physiotherapy programs. The study highlights that when pain is adequately controlled, patients can more actively participate in therapeutic activities, contributing to improved muscle strength and functional mobility (35). Moreover, combining SCS with physiotherapy enhances treatment adherence and quality of life, providing not only pain relief but also increased functional capacity.

Although the benefits of combining SCS and physiotherapy are clear, several difficulties exist in coordinating these treatments. One major barrier is the lack of a clear protocol for integrating the two approaches. Physiotherapy programs need to be tailored to the specific conditions of each SCS-using patient, requiring effective coordination among healthcare professionals. The type of pain, location of the SCS device implantation, and the patient's stage of recovery must all be carefully considered when planning exercises and physical activities (36).

Furthermore, pain perception can vary among patients, and the response to spinal cord stimulation (SCS) may not be uniform. This means that physiotherapy needs to be continuously adjusted to meet the individual needs of each patient. Residual pain or pain in areas not controlled by SCS can interfere with the patient's ability to effectively perform physiotherapy exercises, requiring frequent adjustments in techniques and intensity (37).

Another important challenge relates to patient safety and monitoring during physiotherapy programs. Patients with implanted SCS devices may face restrictions regarding certain types of movements or activities involving the spine (38). High-intensity activities or those involving excessive exertion may cause complications with the implanted device or even affect the effectiveness of the stimulation, thus requiring constant monitoring by physiotherapists.

Despite the challenges, the benefits of combining SCS and physiotherapy are widely recognized. When both treatments are effectively combined, there is a substantial improvement in patients’ quality of life, with pain reduction and increased functional capacity. Patients who followed a combined SCS and physiotherapy regimen experienced significant improvements in mobility, pain control, and autonomy in their daily activities. This combination enables patients to feel more empowered to resume their normal routines and, in many cases, reduce their reliance on analgesics (39).

The integration of SCS and physiotherapy may also result in better long-term outcomes, with a reduction in complications associated with chronic pain, such as muscle atrophy and joint stiffness. Physiotherapy helps maintain muscle function and prevent joint degeneration, which can occur when patients become less active due to chronic pain. SCS, by relieving pain, makes patients more willing to engage in physical activities, which, when combined with physiotherapy, enhances overall recovery (40).

Although many patients indicated for spinal cord stimulation (SCS) have undergone unsuccessful physiotherapy prior to neuromodulation, emerging evidence suggests that physiotherapy introduced after pain relief may enhance long-term outcomes (41, 42). Neuromodulation—whether via SCS or emerging modalities like multifidus muscle stimulation—can reduce pain intensity, enabling patients to better tolerate rehabilitative exercises and potentially improve motor control (43). For example, restorative neurostimulation of the multifidus has shown substantial and sustained improvements in pain, disability, and quality of life, reinforcing the concept of combining neuromodulation with physical rehabilitation (44–47). Additionally, narrative reviews in spinal cord injury rehabilitation have highlighted that combining electrical stimulation with activity-based training results in superior functional recovery compared to either approach alone (46, 48, 49).

Despite this theoretical benefit, none of the clinical studies we reviewed directly compared SCS alone vs. SCS plus structured physiotherapy, and only one included a physiotherapy component—delivered as part of a multidisciplinary rehabilitation program—without isolating its specific contribution to outcomes, as observed in the prospective cohort by Elkholy et al. (13), where improvements in quality of life and reduced opioid use were attributed to SCS, though physiotherapy was also part of patient management. This highlights a critical gap in literature and underscores the need for future trials designed to evaluate the additive or synergistic value of physiotherapy alongside SCS.

In summary, the combination of SCS and physiotherapy in the management of chronic pain offers a promising perspective for the treatment of conditions that do not adequately respond to conventional therapies. SCS, by acting directly on the modulation of pain signals in the spinal cord, has proven effective in reducing chronic pain, especially in cases of persistent low back pain and post-surgical pain syndrome. However, while SCS may provide immediate pain relief, it does not fully resolve the motor and functional impairments often associated with chronic pain. In this context, physiotherapy emerges as an essential complement, enabling functional recovery, muscle strengthening, and improved mobility, promoting a more comprehensive patient rehabilitation.

There is a clear need for more clinical investigations into the effectiveness of SCS combined with physiotherapy. Most existing studies still focus on a single aspect of treatment—either spinal cord stimulation or standalone physiotherapy—with little research addressing the integration of these two therapies and their synergistic effects. For this approach to be more widely adopted and validated, it is essential that more multicenter, controlled, and randomized studies be conducted, with a more detailed analysis of combined treatment protocols, considering different types of chronic pain such as low back pain, post-surgical pain syndrome, and neuropathic pain.

## Data Availability

The raw data supporting the conclusions of this article will be made available by the authors, without undue reservation.

## References

[1] Steingrímsdóttir Ó Landmark A Macfarlane T Nielsen GJ S C. Defining chronic pain in epidemiological studies: a systematic review and meta-analysis. Pain. (2017) 158:2092–107. 10.1097/j.pain.0000000000001009 28767506

[2] Goldberg DS Mcgee SJ. Pain as a global public health priority. BMC Public Health. (2011) 11:770. 10.1186/1471-2458-11-770 21978149 PMC3201926

[3] Manchikanti L Buenaventura RM Manchikanti KN Ruan X Gupta S Smith HS Effectiveness of therapeutic lumbar transforaminal epidural steroid injections in managing lumbar spinal pain. Pain Physician. (2012) 15:E199–245. 10.36076/ppj.2012/15/E199 22622912

[4] Kumar K Taylor RS Jacques L Eldabe S Meglio M Molet J Spinal cord stimulation versus conventional medical management for neuropathic pain: a multicentre randomised controlled trial in patients with failed back surgery syndrome. Pain. (2007) 132:179–88. 10.1016/j.pain.2007.07.028 17845835

[5] Deer TR Mekhail N Provenzano D Pope J Krames E Thomson S The appropriate use of neurostimulation: avoidance and treatment of complications of neurostimulation therapies for the treatment of chronic pain. Neuromodulation appropriateness consensus committee. Neuromodulation. (2014) 17:571–97. discussion 597-578. 10.1111/ner.12206 25112891

[6] Kapural L Yu C Doust MW Gliner BE Vallejo R Sitzman BT Novel 10-kHz high-frequency therapy (HF10 therapy) is superior to traditional low-frequency spinal cord stimulation for the treatment of chronic back and leg pain: the SENZA-RCT randomized controlled trial. Anesthesiology. (2015) 123:851–60. 10.1097/ALN.0000000000000774 26218762

[7] Russo AA Bittner SR Perkins SM Seely JS London BM Lara AH Motor Cortex embeds muscle-like commands in an untangled population response. Neuron. (2018) 97:953–66. e958. 10.1016/j.neuron.2018.01.004 29398358 PMC5823788

[8] Camerota F Petronelli G Savina A Parlangeli MG Truini A Celletti C. Chronic pain: are there effective therapeutic exercises in rehabilitation treatment? A narrative review. Clin Ter. (2024) 175:337–45. 10.7417/CT.2024.5139 39400099

[9] Woolf CJ. Central sensitization: implications for the diagnosis and treatment of pain. Pain. (2011) 152:S2–s15. 10.1016/j.pain.2010.09.030 20961685 PMC3268359

[10] Vicente-Mampel J Falaguera-Vera F Sánchez-Poveda D Hernández-Zaballos F Martinez-Soler M Blanco-Giménez P Spinal cord stimulation combined with exercise in patients diagnosed with persistent spinal pain syndrome. Study protocol for a randomized control trial. PLoS One. (2024) 19:e0309935. 10.1371/journal.pone.0309935 39480792 PMC11527166

[11] Ahmadi SA Vesper J Schu S Slotty PJ. High-Frequency spinal cord stimulation in surgery-naïve patients-A prospective single-center study. Neuromodulation. (2017) 20:348–53. 10.1111/ner.12575 28266756

[12] Al-Kaisy A Palmisani S Smith TE Carganillo R Houghton R Pang D Long-term improvements in chronic axial low back pain patients without previous spinal surgery: a cohort analysis of 10-kHz high-frequency spinal cord stimulation over 36 months. Pain Med. (2018) 19:1219–26. 10.1093/pm/pnx237 29077889

[13] Elkholy MaE Nagaty A Abdelbar AE Simry HaM Raslan AM. Effect of spinal cord stimulation on quality of life and opioid consumption in patients with failed back surgery syndrome. Pain Pract. (2024) 24:261–9. 10.1111/papr.13300 37753793

[14] Gulisano HA Eriksen E Bjarkam CR Drewes AM Olesen SS. A sham-controlled, randomized trial of spinal cord stimulation for the treatment of pain in chronic pancreatitis. Eur J Pain. (2024) 28:1627–39. 10.1002/ejp.2315 38988274

[15] Kallewaard JW Billet B Van Paesschen R Smet I Mendiola A Peña I European Randomized controlled trial evaluating differential target multiplexed spinal cord stimulation and conventional medical management in subjects with persistent back pain ineligible for spine surgery: 24-month results. Eur J Pain. (2024) 28:1745–61. 10.1002/ejp.2306 38943239

[16] Rigoard P Basu S Desai M Taylor R Annemans L Tan Y Multicolumn spinal cord stimulation for predominant back pain in failed back surgery syndrome patients: a multicenter randomized controlled trial. Pain. (2019) 160(6):1410–20. 10.1097/j.pain.0000000000001510 30720582 PMC6553955

[17] Will A Fishman M Schultz D Danko M Verill D Davies C Improvements in therapy experience with evoked compound action potential controlled, closed-loop spinal cord stimulation-primary outcome of the ECHO-MAC randomized clinical trial. J Pain. (2024) 25:104646. 10.1016/j.jpain.2024.104646 39094810

[18] Haack M Simpson N Sethna N Kaur S Mullington J. Sleep deficiency and chronic pain: potential underlying mechanisms and clinical implications. Neuropsychopharmacology. (2020) 45:205–16. 10.1038/s41386-019-0439-z 31207606 PMC6879497

[19] Sturgeon JA Cooley C Minhas D. Practical approaches for clinicians in chronic pain management: strategies and solutions. Best Pract Res Clin Rheumatol. (2024) 38:101934. 10.1016/j.berh.2024.101934 38341332 PMC11512731

[20] Matsuda M Huh Y Ji RR. Roles of inflammation, neurogenic inflammation, and neuroinflammation in pain. J Anesth. (2019) 33:131–9. 10.1007/s00540-018-2579-4 30448975 PMC6813778

[21] Cao B Xu Q Shi Y Zhao R Li H Zheng J Pathology of pain and its implications for therapeutic interventions. Signal Transduction and Targeted Therapy. (2024) 9:155. 10.1038/s41392-024-01845-w 38851750 PMC11162504

[22] Latremoliere A Woolf CJ. Central sensitization: a generator of pain hypersensitivity by central neural plasticity. J Pain. (2009) 10:895–926. 10.1016/j.jpain.2009.06.012 19712899 PMC2750819

[23] Kosek E Clauw D Nijs J Baron R Gilron I Harris R Chronic nociplastic pain affecting the musculoskeletal system: clinical criteria and grading system. Pain. (2021) 162(11):2629–34. 10.1097/j.pain.0000000000002324 33974577

[24] Jensen MP Day MA Miró J. Neuromodulatory treatments for chronic pain: efficacy and mechanisms. Nat Rev Neurol. (2014) 10:167–78. 10.1038/nrneurol.2014.12 24535464 PMC5652321

[25] Jayathilake NJ Phan TT Kim J Lee KP Park JM. Modulating neuroplasticity for chronic pain relief: noninvasive neuromodulation as a promising approach. Exp Mol Med. (2025) 57:501–14. 10.1038/s12276-025-01409-0 40025172 PMC11958754

[26] Fang JY Yamamoto H Romman AN Koutrouvelis A Yamamoto S. Comparative efficacy of spinal cord stimulation in the management of acute pain and chronic pain related to failed back surgery syndrome: a systematic review and meta-analysis of randomized controlled trials. Cureus. (2024) 16(10):e71132. 10.7759/cureus.71132 39525214 PMC11550870

[27] Brill S Defrin R Aryeh IG Zusman AM Benyamini Y. Short- and long-term effects of conventional spinal cord stimulation on chronic pain and health perceptions: a longitudinal controlled trial. Eur J Pain. (2022) 26:1849–62. 10.1002/ejp.2002 35761769 PMC9543320

[28] Leemans L Elma Ö Nijs J Wideman TH Siffain C Den Bandt H Transcutaneous electrical nerve stimulation and heat to reduce pain in a chronic low back pain population: a randomized controlled clinical trial. Braz J Phys Ther. (2021) 25:86–96. 10.1016/j.bjpt.2020.04.001 32434666 PMC7817858

[29] Da Cunha PHM De Andrade DC. The deep and the deeper: spinal cord and deep brain stimulation for neuropathic pain. La Presse Médicale. (2024) 53:104231. 10.1016/j.lpm.2024.104231 38636785

[30] Simopoulos T Sharma S Aner M Gill JS. A temporary vs. permanent anchored percutaneous lead trial of spinal cord stimulation: a comparison of patient outcomes and adverse events. Neuromodulation. (2018) 21:508–12. 10.1111/ner.12687 28901641

[31] Oh S Jekal J Liu J Kim J Park J-U Lee T Bioelectronic implantable devices for physiological signal recording and closed-loop neuromodulation. Adv Funct Mater. (2024) 34:2403562. 10.1002/adfm.202403562

[32] Moens M Goudman L Brouns R Valenzuela Espinoza A De Jaeger M Huysmans E Return to work of patients treated with spinal cord stimulation for chronic pain: a systematic review and meta-analysis. Neuromodulation. (2019) 22:253–61. 10.1111/ner.12797 30117650

[33] Karcz M Abd-Elsayed A Chakravarthy K Aman MM Strand N Malinowski MN Pathophysiology of pain and mechanisms of neuromodulation: a narrative review (A neuron project). J Pain Res. (2024) 17:3757–90. 10.2147/JPR.S475351 39583192 PMC11581984

[34] Solcà M Krishna V Young N Deogaonkar M Herbelin B Orepic P Enhancing analgesic spinal cord stimulation for chronic pain with personalized immersive virtual reality. Pain. (2021) 162:1641–9. 10.1097/j.pain.0000000000002160 33259460

[35] Grider JS Manchikanti L Carayannopoulos A Sharma ML Balog CC Harned ME Effectiveness of spinal cord stimulation in chronic spinal pain: a systematic review. Pain Physician. (2016) 19:E33–54. 10.36076/ppj/2016.19.E33 26752493

[36] Shahali S Shahabi S Etemadi M Hedayati M Anne BC Mojgani P Barriers and facilitators of integrating physiotherapy into primary health care settings: a systematic scoping review of qualitative research. Heliyon. (2023) 9:e20736. 10.1016/j.heliyon.2023.e20736 37860510 PMC10582494

[37] Ahmed SU Zhang Y Chen L St Hillary K Cohen A Vo T Effects of spinal cord stimulation on pain thresholds and sensory perceptions in chronic pain patients. Neuromodulation. (2015) 18:355–60. 10.1111/ner.12316 26033205

[38] Shim JH. Limitations of spinal cord stimulation for pain management. Korean J Anesthesiol. (2015) 68:321–2. 10.4097/kjae.2015.68.4.321 26257842 PMC4524928

[39] Martin SC Baranidharan G Thomson S Gulve A Manfield JH Mehta V Spinal cord stimulation improves quality of life for patients with chronic pain-data from the UK and Ireland national neuromodulation registry. Neuromodulation. (2024) 27:1406–18. 10.1016/j.neurom.2024.06.501 39152988

[40] De La Corte-Rodriguez H Roman-Belmonte JM Resino-Luis C Madrid-Gonzalez J Rodriguez-Merchan EC. The role of physical exercise in chronic musculoskeletal pain: best medicine—a narrative review. Healthcare (Basel). (2024) 12(2):242. 10.3390/healthcare12020242 38255129 PMC10815384

[41] Caylor J Reddy R Yin S Cui C Huang M Huang C Spinal cord stimulation in chronic pain: evidence and theory for mechanisms of action. Bioelectron Med. (2019) 5:12. 10.1186/s42234-019-0023-1 31435499 PMC6703564

[42] Huygen FJPM Soulanis K Rtveladze K Kamra S Schlueter M. Spinal cord stimulation vs medical management for chronic back and leg pain: a systematic review and network meta-analysis. JAMA Network Open. (2024) 7:e2444608–e2444608. 10.1001/jamanetworkopen.2024.44608 39541119 PMC11565267

[43] ElSaban M Kleppel DJ Kubrova E Martinez Alvarez GA Hussain N D'Souza RS. Physical functioning following spinal cord stimulation: a systematic review and meta-analysis. Reg Anesth Pain Med. (2023) 48:302–11. 10.1136/rapm-2022-104295 37080578

[44] Mitchell B Deckers K De Smedt K Russo M Georgius P Green M Durability of the therapeutic effect of restorative neurostimulation for refractory chronic low back pain. Neuromodulation. (2021) 24:1024–32. 10.1111/ner.13477 34242440 PMC8456956

[45] Thomson S Chawla R Love-Jones S Sharma M Vajramani G Williams A Restorative neurostimulation for chronic mechanical low back pain: results from a prospective multi-centre longitudinal cohort. Pain Ther. (2021) 10:1451–65. 10.1007/s40122-021-00307-3 34478115 PMC8586272

[46] Carayannopoulos A Johnson D Lee D Giuffrida A Poply K Mehta V Precision rehabilitation after neurostimulation implantation for Multifidus dysfunction in nociceptive mechanical chronic low back pain. Arch Rehabil Res Clin Transl. (2024) 6:100333. 10.1016/j.arrct.2024.100333 39006113 PMC11240036

[47] Schwab F Mekhail N Patel KV Langhorst M Heros RD Gentile J Restorative neurostimulation therapy compared to optimal medical management: a randomized evaluation (RESTORE) for the treatment of chronic mechanical low back pain due to Multifidus dysfunction. Pain Ther. (2025) 14:401–23. 10.1007/s40122-024-00689-0 39812968 PMC11751280

[48] Singh RE Ahmadi A Parr AM Samadani U Krassioukov AV Netoff TI Epidural stimulation restores muscle synergies by modulating neural drives in participants with sensorimotor complete spinal cord injuries. J Neuroeng Rehabil. (2023) 20:59. 10.1186/s12984-023-01164-1 37138361 PMC10155428

[49] Scheffler MS Martin CA Dietz V Faraji AH Sayenko DG. Synergistic implications of combinatorial rehabilitation approaches using spinal stimulation on therapeutic outcomes in spinal cord injury. Clin Neurophysiol. (2024) 165:166–79. 10.1016/j.clinph.2024.06.015 39033698 PMC11325878

